# Functional Characterization of *Hedychium coronarium* J. Koenig MYB132 Confers the Potential Role in Floral Aroma Synthesis

**DOI:** 10.3390/plants10102014

**Published:** 2021-09-25

**Authors:** Farhat Abbas, Yanguo Ke, Yiwei Zhou, Rangcai Yu, Muhammad Imran, Sikandar Amanullah, Dylan O’Neill Rothenberg, Qin Wang, Lan Wang, Yanping Fan

**Affiliations:** 1 The Research Center for Ornamental Plants, College of Forestry and Landscape Architecture, South China Agricultural University, Guangzhou 510642, China; farhatmerani@yahoo.com (F.A.); keyanguo@126.com (Y.K.); zhouyiwei6333@163.com (Y.Z.); wq2638341289@163.com (Q.W.); WL1361799801@163.com (L.W.); 2 College of Economics and Management, Kunming University, Kunming 650214, China; 3 College of Life Sciences, South China Agricultural University, Guangzhou 510642, China; rcyu@scau.edu.cn; 4 Department of Crop Science and Technology, College of Agriculture, South China Agricultural University, Guangzhou 510642, China; imran_m1303@yahoo.com; 5 College of Horticulture and Landscape Architecture, Northeast Agricultural University, Harbin 150030, China; sikandaraman@yahoo.com; 6 College of Horticulture, South China Agricultural University, Guangzhou 510642, China; Dylan.rothenberg@colorado.edu; 7 Guangdong Key Laboratory for Innovative Development and Utilization of Forest Plant Germplasm, South China Agricultural University, Guangzhou 510642, China

**Keywords:** floral scent, *Hedychium coronarium*, R2R3-MYB, structural genes, terpenes

## Abstract

The R2R3-MYB transcription factors (TFs) play several key roles in numerous plant biological processes. *Hedychium coronarium* is an important ornamental plant well-known for its elegant flower shape and abundant aroma type. The floral aroma of *H*. *coronarium* is due to the presence of a large amount of terpenes and benzenoids. However, less is known about the role of R2R3-MYB TFs in the regulatory mechanism of floral aroma production in this breed. Herein, we isolate and functionally characterize the R2R3-MYB TF HcMYB132, which is potentially involved in regulating floral aroma synthesis. Sequence alignment analysis revealed that it includes a nuclear localization signal NLS(s) and a 2R, 3R motif signature in the sequences. A subcellular localization assay revealed that HcMYB132 protein localizes to the nucleus. Real-time qPCR assays showed that *HcMYB132* is specifically expressed in flowers and its expression pattern correlates with the emission of floral volatile compounds. In *HcMYB132*-silenced flowers, the levels of floral volatile compounds were significantly reduced, and the expression of key structural volatile synthesis genes was downregulated compared to control. Collectively, these results suggest that *HcMYB132* might play a significant role in the regulation of terpenoid biosynthesis in *H. coronarium*.

## 1. Introduction

The floral aroma is one of the crucial characteristics of plants, which improves the economic and aesthetic values of ornamental plants. White ginger lily (*H. coronarium*) is famous due to its pure white color and butterfly flower shape. The *H. coronarium* flower emits a strong aroma, which is a combination of several floral volatiles including terpenes, benzenoids, and phenylpropanoids [1,2,3,4,5]. Monoterpenes and sesquiterpenes are the major floral volatile contents of this breed, and in our previous studies we identified several key volatile synthesis genes (*HcTPS1/2/3/5/7/8/10*, *HcBSMT1/2*, *HcIAA2/4*, *HcARF5* and *HcPAL*) involved in floral aroma biosynthesis [6,7,8,9]. The identification of the genes, transcription factors (TFs), and proteins relevant to floral scent biosynthesis has been advanced. However, less is known about the regulatory mechanism of R2R3-MYB TFs in *H*. *corornarium*. In our previous RNA sequence and genome-wide data, we reported on a group of *HcMYB* genes potentially involved in the regulating mechanism of secondary metabolites [1,10]. Among them, *HcMYB132* is specifically expressed in flowers and its expression correlates with flower development and emission contents of floral volatiles. However, a detailed functional characterization of this transcription factor in *H*. *coronarium* has not yet been produced.

MYB TFs are vital regulators of secondary metabolites such as isoflavones and phenylpropanoids [11,12,13]. MYB TFs are classified into four groups based on the number of repeats (1R, R2R3, 3R, and 4R-MYB) [13]. Among them, R2R3-MYB domain proteins are widely abundant in plants and play important role in several processes, including environmental stress, growth and development, secondary wall biosynthesis, and flavonoid/phenylpropanoid metabolism [14,15,16,17]. For example; *GbMYB5*, *AtMYB44* and *AtMYB60* induced drought tolerance in cotton and *Arabidopsis* [18,19]. *AtMYB33* and *AtMYB65* assist in the formation of viable pollen and produce high pollen fertility, while *AtMYBL2* functions as a transcriptional repressor, and prevents the accumulation of proanthocyanin in *Arabidopsis* [12,20]. In *Malus domestica*, *MdMYB3* modulates the production of anthocyanin via its effect on the various flavonoid pathway genes and assists in flower formation [21]. Similarly, *Arabidopsis AtMYBL2/4/7* and litchi R2R3-MYB showed their important role in the regulation of flavonoid and anthocyanin biosynthesis, respectively [12,22,23]. The soybean *GmMYB100*-and grape *VvMYB4*-like genes negatively regulate the production of flavonoids [24,25]. 

However, only limited MYB TFs related to volatile biosynthetic pathways have been characterized from a few plant species, including snapdragon (*Antirrhinum majus*) and petunia (*Petunia* spp.), which are known as model floral scent species. The volatile phenylpropanoid/benzenoid metabolic pathway is regulated by *AmMYB305/340*, ODORANT 1 (*ODO1*), and EMISSION OF BENZENOID II (*EOBII*) in snapdragon [26,27] and petunia, respectively [28,29,30]. Likewise, *PpMYB15* and *PpMYBF1* exhibited a floral expression and participated in the biosynthetic control of flavanol from *Prunus persica* [31]. The production of phenylalanine and its metabolic flow to lignin biosynthesis are controlled by *MYB8* and ELONGATED HYPOCOTYL (*HY5*) in *Pinus pinaster* [32]. Until now, several reports of MYB TFs related to flavonoid biosynthesis in other species have been discussed, but still, there is a gap in knowledge of the role of MYB in *H. coronarium*. 

In the current study, *HcMYB132* was isolated and functionally characterized. Multiple sequence analyses revealed the existence of 2R and 3R motif signatures in the sequences. Furthermore, qRT-PCR, green fluorescent protein (GFP), hormone treatments, GC-MS, and gene silencing assays were performed to elucidate its potential involvement in floral aroma biosynthesis in *H. coronarium*. These findings will lay the cornerstone for the functional characterization of MYB TFs in *H. coronarium*.

## 2. Results

### 2.1. Characterization of HcMYB132 

**Figure 1:**
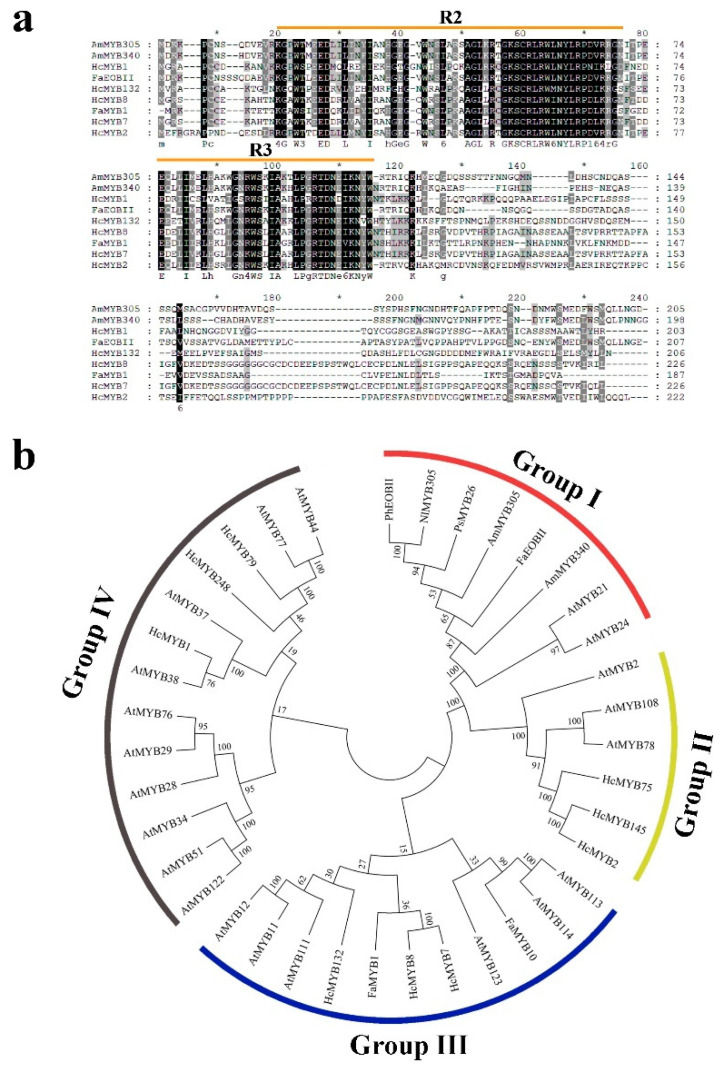
Sequence alignment and phylogenetic analysis of HcMYB132. (**a**) Multiple sequence alignment of HcMYB132 with R2R3-MYB proteins. Sequence alignment was performed by ClustalX 2.1 and shaded in GeneDoc. Amino acid residues are shaded in light gray, gray, and black showing 50, 70 and 100% identity, respectively, while dashes indicate gaps used for optimal alignment. R2R3 motifs are indicated by orange lines. (**b**) Phylogenetic analysis of HcMYB132 together with previously characterized R2R3-MYB proteins. The protein sequences were aligned by Clustal X 2.1 and the phylogenetic tree was built in MEGA X using the Nj method. All R2R3-MYBs are grouped into 4 subclades named G I–G IV. Genes used in phylogenetic tree and their accession numbers are listed in Table S2.

In a previous genome-wide analysis, we identified a group of R2R3-MYB family members expressed specifically in flowers that increased in expression with flower development and floral volatile emissions [1]. Among them, *HcMYB132* is specifically expressed in flowers. The coding sequences of *HcMYB132* include open reading frames of 624 bp, encoding polypeptides of 207 amino acid residues with a molecular weight of 23.76 kilodaltons (kDa), isoelectric point (*pI*) 6.16, and the protein GRAVY −0.733. Further analysis revealed that *HcMYB132* contains two exons, and is located on chromosome 11. Prediction analysis of HcMYB132 protein sequences showed the presence of R2 and R3 repeat signatures at the N-termini, which is a key feature of R2R3 DNA-binding MYB proteins (Figure 1a). 

The phylogenetic analysis of HcMYB132 was performed with the previously characterized R2R3-MYB proteins involved in secondary metabolism derived from *H*. *coronarium* and other plant species. All R2R3-MYBs were clustered into 4 distinct groups (G I–G IV) (Figure 1b). Among them, subgroup G II included the least number of R2R3-MYB members (6), while subgroup G IV constituted the largest group, holding 13 R2R3-MYB members. HcMYB132 clustered into subgroup III, which included FaMYB1/10 (*Fragaria* × *ananassa*), HcMYB7/8 (*H*. *coronarium*), and AtMYB11/12/111/113/114/123 (*Arabidopsis thaliana*). 

### 2.2. Subcellular Localization of HcMYB132

**Figure 2:**
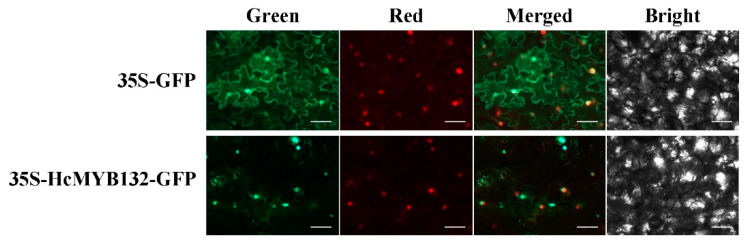
Nuclear localization of *H*. *coronarium* MYB132 protein in *N*. *benthamiana* leaves. Green: GFP fluorescence, red: mcherry as NLs marker, merged: merged green and red channels and bright field. Bars, 50 µM.

Nuclear localization prediction tools predicted that HcMYB132 is located in the nucleus. To verify the prediction results, we generated HcMYB132-GFP constructs driven by a CaMV *35S* promoter and transferred them to *N*. *benthamiana* leaves via agroinfiltration, followed by visualization using confocal laser scanning microscopy (Zeiss, Jena, Baden-Württemberg, Germany). The results verified that HcMYB132 protein was localized to the nucleus (Figure 2).

### 2.3. Expression Pattern of HcMYB132 

**Figure 3:**
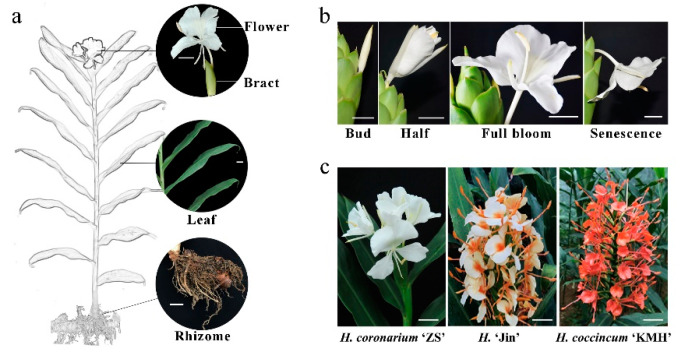
A pictorial view of labeled *H*. *coronarium* tissues. (**a**) Figure representation of *H*. *coronarium* flower, bracts, leaves, and rhizome; (**b**) figure illustration of different flower developmental stages (bud stage, half bloom, full-bloom and senescence stage); (**c**) pictorial representation of three different *Hedychium* accessions. Scale bar indicates 2 cm.

**Figure 4:**
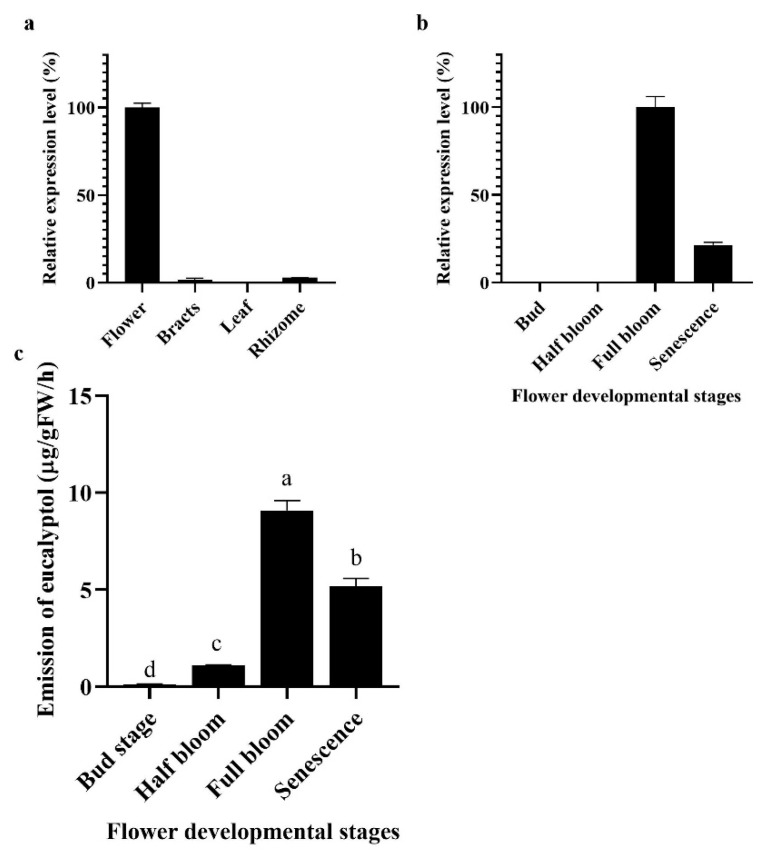
Expression analysis of *HcMYB132* in different tissues. (**a**) Relative expression level of *HcMYB132* in different parts; (**b**) different flower development stages of *H*. *coronarium,* results are shown as a percentage with a maximum value set to 1 (100%); (**c**) emission level of eucalyptol during flower development stages, data are shown as ± SEM of three to five repeats. Lowercase letters represent statistically significant differences at *p* < 0.01, according to least significant difference (LSD).

Previous research indicated that the accumulation of floral volatiles increases with flower development [1,2,7]. To analyze the aforementioned process, flower development was divided into four stages (Figure 3 and Figure 4). 

The data showed that *HcMYB132* was specifically expressed in flowers, while negligible expression was measured in the rhizome and bracts (Figure 4a). Furthermore, the mRNA transcript levels of *HcMYB132* were abundant in the full-bloom stage, and low during senescence (Figure 4b). A similar pattern was observed in the emission level of eucalyptol contents; low during the bud stage, peaking during full bloom, and decreasing thereafter (Figure 4c).

### 2.4. Suppression of HcMYB132 Modifies the Emission of Eucalyptol and Expression of Key Structural Genes

**Figure 5:**
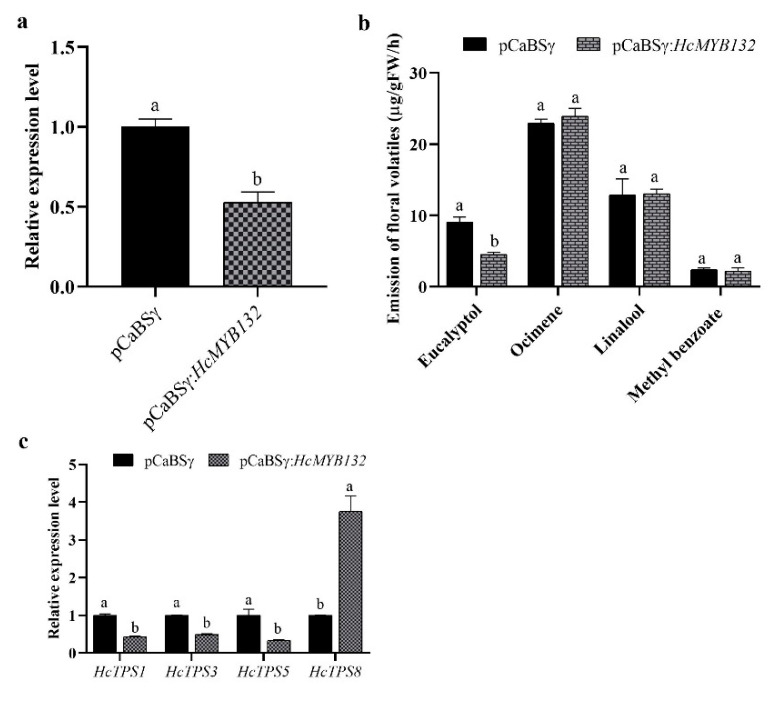
Suppression of *HcMYB132* in *H. coronarium* flowers. (**a**) RT-qPCR assay of *HcMYB132* transcript levels in *HcMYB132*-silenced and control flowers; (**b**) GC-MS analysis of floral volatiles in *HcMYB132*-silenced and control flowers; (**c**) transcript levels of key structural genes in *HcMYB132*-silenced and control flowers. Data are shown as ± SEM of three to five repeats. Lowercase letters represent statistically significant differences in LSD test (*p* < 0.01).

The results confirmed that the expression level of *HcMYB132* was significantly reduced compared to the control (unsilenced flowers) (Figure 5). Transcript levels of *HcMYB132* were downregulated by 47.42% in silenced flowers compared to the control flowers (Figure 5a). Furthermore, the volatile contents of eucalyptol were decreased by 50% in response to *HcMYB132* silencing, while ocimene, linalool, and methyl benzoate contents did not significantly change (Figure 5b). 

We further investigated the mRNA level of the key genes involved in eucalyptol biosynthesis (*HcTPS1*, and *HcTPS3*) and few other *HcTPSs* (*HcTPS5* and *HcTPS8*) in *HcMYB132*-silenced flowers. The results revealed that transcript levels of *HcTPS1*, *HcTPS3* and *HcTPS5* were significantly decreased, while the expression level of *HcTPS8* was significantly increased. In *HcMYB132*-silenced flowers, *HcTPS1*, *HcTPS3*, and *HcTPS5* transcripts were all significantly reduced, by 56.45, 50.44, and 65.90%, respectively, compared to control (Figure 5c). Interestingly, the mRNA levels of *HcTPS8* increased by 275.72% compared to the control, implying that *HcMYB132* positively regulates the expression of *HcTPS1*, *HcTPS3,* and *HcTPS5*, and negatively regulates the expression of *HcTPS8*. These findings indicate that *HcMYB132* plays a significant role in floral aroma production in *H*. *coronarium*. 

### 2.5. Expression of HcMYB132 in Response to Auxin and PCIB Treatments

**Figure 6:**
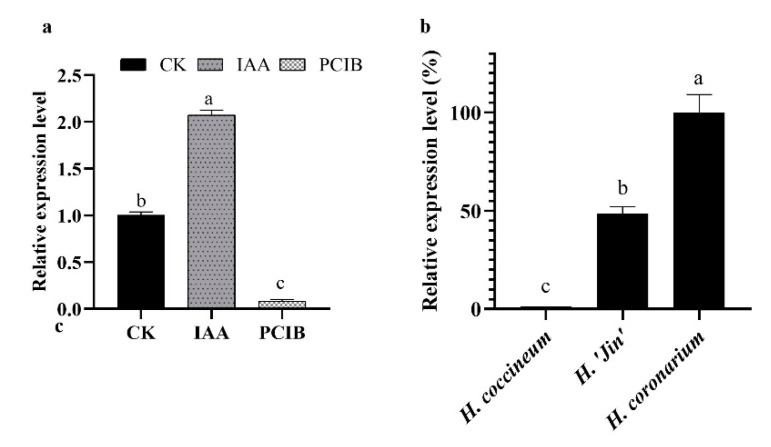
mRNA levels of *HcMYB132* and emission level of eucalyptol under IAA and PCIB treatment. (**a**) transcript levels of *HcMYB132* under IAA and PCIB treatment; (**b**) mRNA levels of *HcMYB132* in IAA and PCIB treated flowers. Error bars indicate SD of 3–5 repeats, and lowercase letters indicate significant differences using the LSD test (*p* < 0.01).

The treatment results showed that the expression level of *HcMYB132* was substantially increased in response to IAA treatments (Figure 6a). Under IAA treatments, the expression level of *HcMYB132* increased by 107.45% compared to the control flowers. In a previous study, we found that under IAA treatment the emission contents of eucalyptol significantly increased [7], and the emission contents of eucalyptol were also found to increase by 102.35% under IAA treatment, relative to the untreated control.

To validate the above findings, we examined the same parameters under PCIB. PCIB has extensively been used to inhibit the actions of auxin. The results showed that the transcript levels of *HcMYB132* decreased substantially, by 91.73%, in PCIB treated flowers compared to flowers not treated with PCIB (Figure 6a). Likewise, under PCIB treatment, eucalptol emission contents declined significantly, by 64.04%, in PCIB treated flowers compared to the control [7]. These findings support the above-mentioned results that *HcMYB132* significantly influenced the biosynthesis of floral aroma production via auxin signaling. 

We further analyzed the expression level of *HcMYB132* in three different Hedychium accessions. The data showed that the transcript levels of *HcMYB132* were highest in *H*. *coronarium* followed by *H*. ‘Jin’ and *H*. *coccineum*, respectively (Figure 6b). These results suggest that *HcMYB132* is potentially involved in floral aroma production in *H*. *coronarium.*

## 3. Discussion

*H*. *coronarium* is popular in tropical and subtropical parts of the world due to its appealing strong aroma type and medicinal properties [3,33]. R2R3-MYB TFs are the main regulators of terpenes and phenylpropanoids [34,35]. However, less is known about the transcriptional regulatory mechanism of floral aroma production. Until now, a few MYB TFs have been reported that control the regulatory network of floral scent production [29,30,36,37]. Herein, we identified and functionally characterized a R2R3-MYB TF (HcMYB132) that is potentially involved in floral aroma synthesis in *H*. *coronarium*. 

Multiple sequence analyses of HcMYB132 revealed the existence of 2R and 3R repeats in the sequences (Figure 1a). Several previous findings suggest that the R2 and R3 signature motifs are highly conserved and regulate various aspects of plant secondary metabolites [13,38,39,40]. We generated a phylogenic tree using the previously characterized R2R3-MYB TFs involved in the regulatory network of secondary metabolism, together with HcMYB132 (Figure 1b). HcMYB132 was classified into Group III with FaMYB1, FaMYB10, and AtMYB11/12/111/113/114/123. The functional characterization of aforementioned genes revealed their role in the regulation of the flavonoid/phenylpropanoid metabolism [14,41,42,43], indicating that *HcMYB132* might play a significant role in secondary metabolism. It has been reported that MYB TFs in same subclade have identical functions [13,35]. The structure analysis revealed that the *HcMYB132* contains two exons, which are in line with the previous reports [44]. A subcellular localization assay revealed that HcMYB132 protein is localized to the nucleus, which is consistent with the previous findings [1,7,13,45]. 

The process of floral scent production is interrelated with flower development [46,47,48]. Our previous studies revealed that production and emission of floral volatile compounds and the expression of key structural volatile biosynthesis genes were low during the bud stage and peaked during the full bloom stage [7,8,9,10]. Previous studies also showed that volatile emission content was significantly larger from the flower than from the rhizome and leaf, which is consistent with the expression pattern of *HcMYB132* [7]. In the current findings, it was revealed that *HcMYB132* was mainly expressed in the flowers and its expression pattern increased with flower development, peaked during the fully bloomed stage, and dropped down thereafter (Figure 4a,b), implying that it might potentially be involved in the floral aroma production and emission mechanism. A similar expression pattern was observed in *Fragaria ananassa EOBII*, *EOBI*, and *ODO1*, and was involved in the regulatory network of eugenol [15,29]. Likewise, *Prunus persica MYBF1* and *MYB15* showed the highest expression in the flower and were involved in flavanol biosynthesis regulation [31]. In *Lilium hybrid*, ODO1 TF had highest expression in the flower and plaedy a crucial role in the regulation of phenylpropanoid/ benzenoid volatile production [49]. These results suggest that *HcMYB132* potentially regulates the process of floral scent production.

To reveal the role of *HcMYB132* in floral aroma production in *H*. *coronarium*, the activity of *HcMYB132* was repressed via gene silencing. The data showed that the volatile contents of eucalyptol were substantially decreased in *HcMYB132*-silenced flowers compared to control flowers. Furthermore, in *HcMYB132*-silenced flowers, the transcript levels of key eucalyptol volatile biosynthesis genes (*HcTPS1* and *HcTPS3*) were significantly decreased (Figure 5). Likewise, strawberry *MYB10* regulates the expression of numerous key genes involved in the flavonoid and phenylpropanoid biosynthesis process [14]. In petunia *ODO1*-suppressed plants, the mRNA levels of several scent-related genes were downregulated [29]. Similarly, litchi *MYB5* activates the transcript levels of key genes involved in the synthesis of anthocyanin [23]. In *HcMYB1/2/7/8/75/79/145/238/248*-silenced flowers, the emission of floral volatiles and the expression of structural genes were significantly decreased [1,7]. Moreover, the emission of eucalyptol and the expression of *HcMYB132* were influenced by auxin treatments, which are consistent with previous findings [7,50]. These data endorse the previous findings that R2R3-MYB TFs are involved in the regulation of volatile formation in *H*. *coronarium*.

## 4. Materials and Methods

### 4.1. Plant Materials and Growth Conditions

Plants (*H. coronarium*, *H. coccincum*¸and *H*. ‘Jin’) were planted in a growth chamber at 25 ± 2 °C with 75–80% humidity and a 13 h–11 h light-dark cycle. To analyze the spatial and temporal expression pattern, different plant parts including the rhizome, flower, leaf and bracts of *H*. *coronarium* were used (Figure 3a). To analyze the expression pattern of *HcMYB132* during *H*. *coronarium* flower development, the flower developmental process was divided into four stages; bud, half bool, full bloom, and senescence (Figure 3b). For RNA isolation, plant materials were obtained and immediately frozen in liquid nitrogen, then stored at −80 °C. For the subcellular localization assay, *N. benthamiana* plants were grown under the same conditions. The plant materials were remained in the growth chamber until analysis.

### 4.2. Hormone Treatments

For hormone treatment, the stems of the *H*. *coronarium* flowers were cut into 40 cm section, and placed in sterilized water comprising 100 μM IAA and 100 μM p-chlorophenoxyisobutyric acid (PCIB). IAA and PCIB stock solutions (Sigma-Aldrich, St. Louis, Missouri, United States) were made following the manufacturer’s instructions. In short, IAA (18.79 mg) powder was liquified in 1.5 mL methanol and then diluted in sterilized water (100 mL). Similarly, PCIB powder (321 mg) was dissolved and diluted as mentioned above. Afterward, detached flowers were put in glass beakers that included the hormone solution (100 mL), and covered with a silver sheet to prevent degradation. The mock/control flowers were placed under the same conditions and same volume as described above. The volatile compound analysis was carried out at the full-bloom stage of treated flowers, which were subsequently frozen in liquid nitrogen and stored at –80 ℃. The experiment was performed in triplicate for each experimental variant. 

### 4.3. Bioinformatics Analysis

The sequence of *HcMYB132* was obtained from the previously published MYB genome-wide data [1]. The other scent-related protein sequences were obtained from the NCBI database. The amino acid sequences were aligned using Clustal Ω [51], and a phylogenetic tree was generated in MEGA X [52] by selecting the neighbor-joining (NJ) method with 1000 bootstrap replicates. 

### 4.4. Subcellular Localization Analysis 

For subcellular localization, Hthe cMYB132 coding sequence with *SpeI* and *NcoI* restriction sites was fused into the vector pEAQ-HT-GFP [53]. The ClonExpress ^®^ II one-step cloning kit (Vazyme, China) was used to construct the vectors. Sequencing confirmed that no errors had been introduced. The plasmid was introduced into *Agrobacterium tumefaciens* (strain EHA105) and Luria–Bertani (LB) medium with antibiotics, then was cultured overnight. After that, pellets were collected via centrifugation at 2000× *g* and resuspended in MMA solution (10 mM MgCl2, 100 μM acetosyringone, 10 mM MES (2-[N-morpholino] ethane sulfonic acid) with pH 5.8 to an OD600 of 0.6. The suspension was infiltrated into *N*. *benthamiana* leaves as explained previously [2,8]. The infected tissues were visualized 48 h after infiltration by a Leica TCS SP2 AOBS spectral confocal scanner mounted on a Leica DM RXA2 upright fluorescence microscope with 409 × 0.75 numerical aperture objectives, and the images were further processed using Adobe Photoshop. Primers used in the assay are listed in Table S1.

### 4.5. Virus-Induced Gene Silencing (VIGS)

To analyze the potential role of *HcMYB132* in floral aroma production, we suppressed its expression via virus-induced gene silencing (VIGS) in flowers. For VIGS, A 250–300 bp amplicon of *HcMYB132* gene was inserted in a pCaBSγ vector using Apa I as a restriction site, making a pCaBSγ:*HcMYB132* construct for the silencing of the corresponding gene, as described previously [54]. The constructs (pCaBS-α, pCaBS-β, pCaBSγ, pCaBSγ:*HcMYB132*) were transformed into *Agrobacterium tumefaciens* (EHA105). The transformed *A. tumefaciens* lines were cultured in LB medium supplemented with 50 μg/mL kanamycin and 25 μg/mL rifampicin. The cultures were harvested by centrifugation at 5000 rpm for 10 min and resuspended in infiltration buffer (10 mM MgCl2, 0.1 mM acetosyringone, 10 mM MES, pH 5.6). For infiltration, *A. tumefaciens* culture was suspended in infiltration buffer to an OD600 of 1. The solution was applied at the bud stage by vacuum infiltration via submerging the flowers in the bacterial solution. The culture mixtures were placed at room temperature in the dark for 3 to 5 h before vacuum infiltration into the *H*. *coronarium* flowers. Thereafter, the flowers were cleaned with deionized water and placed into an MS liquid culture at 16 °C with a 12/12 h light/dark cycle for 4–5 days. The floral volatile analysis was performed during the full-bloom stage via GC–MS. The assay was carried out in 3–5 biological replicates.

### 4.6. GC-MS Analysis of Floral Volatiles 

The floral volatile analysis was performed by placing the whole flower in a glass bottle for 30 min, as explained previously [7,55]. Polydimethylsiloxane (PDMS) fiber was inserted into the bottle for 30 min to adsorb volatile compounds followed, then injected into a GC-MS system (Agilent). The GC–MS system with Agilent 7890A GC and Agilent 5975C MSD was provided with an Agilent DB-5MS capillary column (30 m × 0.25 mm), and helium gas was provided as a carrier. The flow of helium gas was kept constant at 1 mL/min. Initially, the GC injection port temperatures were kept at 40 °C for 3 min, which was followed by an increase in temperature of 5 ℃/min to 250 °C. The chromatographic running time was 30 min. The relative quantification of volatiles was calculated using the Agilent ChemStation data analysis application based on the peak area ratio and the quantity of the internal standard.

### 4.7. Identification of Floral Volatiles

The floral volatile compounds were identified by comparing them with mass spectra from the NIST mass spectral library (NIST 08), with existing works of literature, and with authentic standards. Mass spectra were obtained by automatic scanning at m/z 20 to 500 amu. The identification of compounds was perceived via comparing the mass spectra with NIST 08 at a match factor of ≥80. The data were processed using mass hunter qualitative analysis workflow software (Agilent Technologies Inc., Santa Clara, CA, USA).

### 4.8. RNA Isolation, cDNA Synthesis, and RT-qPCR

Total RNA isolation and cDNA was synthesized as explained earlier [56,57]. Total RNA from different organs/tissues and flower developmental stages was extracted using a HiPure plant RNA mini kit (Magen, Guangzhou, China) according to the manufacturer’s suggestions. In total RNA, genomic DNA contamination was removed by DNase I. The qRT-PCR analysis was executed in an ABI 7500 fast real-time PCR system (Applied Biosystems, MA, USA) using iTaqTM Universal SYBR Green Supermix (BIO-RAD, CA, USA) following the manufacturer’s protocols. PCR was performed in a total volume of 20 µL containing 10 µL iTaq ™ Universal SYBR Green Supermix (BIO-RAD), 7.2 µL of ddH_2_O, 0.4 µL each of forward and reverse primers, and 2 µL of cDNA, using an ABI 7500 Fast Real-Time PCR System (Applied Biosystems, USA). GAPDH was used for normalization of data and the 2 °C^-ΔΔ*C*T^ method was employed for measuring the relative expression analysis [58]. The reactions were performed in triplicate. 

### 4.9. Data Analysis

Statistical Package for the Social Sciences 19.0 (SPSS Inc., Chicago, IL, USA) was used for the statistical analysis. The differences among samples were calculated via analysis of variance (ANOVA). Data are presented as the mean ± SD (*n* = 3–5).

## 5. Conclusions

In the present study, an R2R3-MYB TF (*HcMYB132*) was isolated and functional characterized. Expression pattern analysis revealed that *HcMYB132* was highly expressed in the flowers and its expression pattern correlated with flower development and emission of floral volatiles and was influenced by auxin. Suppression of *HcMYB132* resulted in the downregulation of key structural genes and a decreased emission level of eucalyptol contents. Subcellular localization assay showed that HcMYB132 was localized to the nucleus. 

## Data Availability

The data presented in this study are available in article and supplementary material.

## Supplementary Materials

The following are available online at https://www.mdpi.com/article/10.3390/plants10102014/s1, Table S1: Primers used in the experiments, Table S2: Genes used in phylogenetic tree and their accession numbers.
