# Supplementary material for: Functional Characterization of Hedychium coronarium J. Koenig MYB132 Confers the Potential Role in Floral Aroma Synthesis
Source: Plants (Basel). 2021 Sep 25;10(10):2014. doi: 10.3390/plants10102014 (PMC8541032; doi:10.3390/plants10102014)
Supplement: Supplementary file 1 [file plants-10-02014-s001.zip › plants-1400724-supplementary.pdf]

Table S1: Primers used in the experiments

**Cloning primers**		Upstream / downstream primers （5′-3′）
HcMYB3	F	GAAGCACCTTCTTGCTCAGTG
	R	GAAGCACCTTCTTGCTCAGTG
**qRT-PCR primers**		
HcMYB3	F	GAAGAAGAAGAGTTTCTTC
	R	GAAGAAGAAGAGTTTCTTC
**BSMV**		
HcMYB3	F	AAGGAAGTTTAAGCCCAAACATGCAATTACCAG
	R	AAGGAAGTTTAAGCCCAAACATGCAATTACCAG
**GAPDH**		
	F	GGTATTGTCGAGGGTTTGATG
	R	GCTGTTGGCAAAGTTCTCCCT
**GFP primers**		
HcMYB3	F	CAAATTCGCGACCGGT ATGGTGAGAGCTCCTTG
	R	CAAATTCGCGACCGGT ATGGTGAGAGCTCCTTG

Table S2: Genes used in phylogenetic tree and their accession numbers.

**No.**	**Gene name**	**Plant Name**	**Accession number**
1	FaEOBII	*Fragaria x ananassa*	KM099230
2	AmMYB305	*Antirrhinum majus*	P81391
3	AmMYB340	*Antirrhinum majus*	P81396
4	PsMYB26	*Pisum sativum*	Y11105
5	PhEOBII	*Petunia hybrida*	EU360893
6	NlMYB305	*Nicotiana langsdorffii*	EU111679
7	FaMYB1	*Fragaria x ananassa*	AF401220
8	FaMYB10	*Fragaria x ananassa*	EU155162
9	AtMYB11	*Arabidopsis thaliana*	AT3G62610
10	AtMYB12	*Arabidopsis thaliana*	AT2G47460
11	AtMYB123	*Arabidopsis thaliana*	AT5G35550
12	AtMYB111	*Arabidopsis thaliana*	AT5G49330
13	AtMYB113	*Arabidopsis thaliana*	AT1G66370
14	AtMYB114	*Arabidopsis thaliana*	AT1G66380

15	AtMYB21	*Arabidopsis thaliana*	AT3G27810
16	AtMYB24	*Arabidopsis thaliana*	AT5G40350
17	AtMYB2	*Arabidopsis thaliana*	AT2G47190
18	AtMYB108	*Arabidopsis thaliana*	AT3G06490
19	AtMYB78	*Arabidopsis thaliana*	AT5G49620
20	AtMYB122	*Arabidopsis thaliana*	AT1G74080
21	AtMYB51	*Arabidopsis thaliana*	AT1G18570
22	AtMYB34	*Arabidopsis thaliana*	AT5G60890
23	AtMYB29	*Arabidopsis thaliana*	AT5G07690
24	AtMYB76	*Arabidopsis thaliana*	AT5G07700
25	AtMYB28	*Arabidopsis thaliana*	AT5G61420
26	AtMYB38	*Arabidopsis thaliana*	AT2G36890
27	AtMYB37	*Arabidopsis thaliana*	AT5G23000
28	AtMYB77	*Arabidopsis thaliana*	AT3G50060
29	AtMYB44	*Arabidopsis thaliana*	AT5G67300
